# Impact of long-term cryopreservation on serum proteome and metallome: Implications for Biobank quality control

**DOI:** 10.1371/journal.pone.0351736

**Published:** 2026-06-25

**Authors:** Meng Lu, Xiongshun Liang, Wanna Xu, Hongxing Tan, Junan Yan, Xuqiao Hu, Wenxu Hong

**Affiliations:** 1 Shenzhen Center for Chronic Disease Control (Shenzhen Dermatology Hospital), Shenzhen, China; 2 Shenzhen Institute of Dermatology, Shenzhen, China; 3 Shenzhen Center for Chronic Disease Control Biobank, Shenzhen, China; Cardiff Metropolitan University, UNITED KINGDOM OF GREAT BRITAIN AND NORTHERN IRELAND

## Abstract

Biobanks constitute a cornerstone of modern biomedical research, yet the effects of long-term storage on biospecimen integrity are not fully characterized. This study systematically evaluated the impact of prolonged cryopreservation on the metallomic and proteomic profiles of human serum. We analyzed 400 serum samples from the Shenzhen Chronic Disease Risk Factor Surveillance Project, including 200 long-term and 200 short-term cryopreserved specimens from healthy adults. Inductively coupled plasma mass spectrometry (ICP-MS) was used to quantify ten metals: vanadium (V), chromium (Cr), manganese (Mn), iron (Fe), copper (Cu), zinc (Zn), selenium (Se), rubidium (Rb), strontium (Sr), and cesium (Cs). Proteomic profiling was performed via liquid chromatography-tandem mass spectrometry (LC-MS/MS). The analysis quantified 682 proteins, with 242 showing significant abundance changes (fold change > 1.5, P < 0.05; 176 up-regulated, 66 down-regulated). Functional enrichment revealed these changes were linked to immune response, metabolic pathways, and antioxidant activity (P < 0.05). Concentrations of V, Mn, Fe, Zn, Rb, and Cs were significantly altered in long-term versus short-term stored samples (*P* < 0.05). Moreover, long-term cryopreservation substantially reshaped the inter-element correlation networks. In summary, prolonged storage significantly modifies the serum metallome and proteome, potentially affecting the validity of research conclusions derived from biobanked materials. Consequently, storage duration must be rigorously considered and adjusted for in the design and interpretation of future large-scale retrospective studies utilizing archived specimens.

## Introduction

Biobanks serve as the cornerstone for advancing modern medicine, as the high-quality biological samples they preserve provide indispensable resources for the discovery of disease biomarkers, the innovation of clinical diagnostics, and the development of therapeutic strategies [1]. As biobanks continue to expand in scale, ensuring the molecular integrity of samples during long-term storage has become a critical issue in biobank science. Sample quality is influenced by various factors throughout the entire process of collection, processing, and storage, among which key variables such as storage temperature, freeze-thaw cycles, and storage duration have been shown to significantly alter the molecular composition and analytical reliability of biological samples [2–4].

Taking major global biobanks as an example, the total number of samples preserved has exceeded 300 million, with tens of millions of new samples added annually. However, this growth in scale is accompanied by alarming quality concerns: nearly half of researchers report difficulties in obtaining samples that meet analytical standards in practical work, over 60% express reservations about the accuracy of results obtained from long-term stored samples, and more than 80% of scholars agree that the scarcity of high-quality samples has become a critical bottleneck restricting research progress [5]. Although international organizations have established systematic quality control guidelines and introduced standardized protocols, such as the Standard PREanalytical Code (SPREC) [6–8], the specific mechanisms and extent of the systemic impact of long-term cryopreservation on two key categories of biomolecules—the proteome and metallome—remain insufficiently clear.

Current research on storage stability has predominantly focused on macro metal elements such as sodium, potassium, calcium, and iron [9–14]. While these studies provide important references for understanding the storage behavior of basic metals, the scope of investigation is notably limited. Importantly, despite their extremely low in vivo concentrations, metal elements such as chromium, manganese, and selenium play irreplaceable roles in regulating oxidative stress, maintaining neurological function, and ensuring metabolic homeostasis. They are key actors in the pathogenesis and progression of neurodegenerative diseases, cardiovascular disorders, and environmentally related illnesses [15,16]. If long-term frozen storage alters the true concentrations of these metals, the reliability of disease associations drawn from stored samples could be fundamentally compromised.

To systematically elucidate the effects of long-term frozen storage on biological samples—specifically the changes in metallomic concentrations and protein profiles of long-term cryopreserved serum samples—this study employed inductively coupled plasma mass spectrometry (ICP-MS) for metal quantification and liquid chromatography-tandem mass spectrometry (LC-MS/MS) with data-independent acquisition (DIA) for proteomic analysis. The aim is to clarify the influence of cryostorage duration on the stability of serum metals and to provide a reference for data correction in future large-scale retrospective studies.

## Materials and methods

### Study population and storage conditions

Serum samples analyzed in this study originated from the biobank of the Shenzhen Center for Chronic Disease Control. These specimens were initially collected as part of the Shenzhen Chronic Disease Risk Factor Surveillance Project, a community-based health survey conducted in 2018 and 2023 to profile local metabolic risk factors. All blood draws were performed using 5 mL serum‑separating vacuum tubes. Upon arrival at the laboratory under refrigerated conditions (4 °C), each sample was centrifuged at 3000 rpm/min for 15 minutes, then aliquoted and stored at –80 °C on the same day. This study protocol was reviewed and approved by the Ethics Committee of the Shenzhen Center for Chronic Disease Control (Approval No.: SZCCC‑2024‑047‑01‑PJ), and written informed consent was obtained from every participant prior to sample collection. Patient records, de-identified and assigned unique codes, were provided to the research team and accessed for analysis in December 2024.

To establish a metabolically stable reference cohort, we enrolled healthy Han Chinese adults aged 18–33 years, balancing gender equally (1:1 male‑to‑female ratio) and restricting body mass index (BMI) to 18–24 kg/m^2^. Each potential participant completed a standardized health screening before enrollment to confirm the absence of diagnosed disease or abnormal clinical indicators.

The selection process further excluded specimens that could compromise analytical reliability. This included samples showing visible hemolysis or lipemia, those with documented freeze‑thaw cycles, and any lacking complete storage documentation. Individuals with a known history of chronic diseases—such as hypertension, diabetes, or cardiovascular disorders—were also excluded, ensuring that the final sample set reflected a generally healthy population without confounding metabolic conditions.

### Proteomic analysis

Serum samples from 30 healthy individuals from the long-term cryopreservation group and 30 age- and sex-matched healthy individuals from the short-term cryopreservation group were included. Within each group, samples were randomly divided into three biological pools, each containing ten distinct individual samples. Each pool was processed and analyzed independently as a biological replicate. Proteins (150 µg per sample) were processed using the High-Select™ Top 14 Abundant Protein Depletion Kit (Thermo Fisher Scientific, USA) for the removal of high-abundance proteins. Reduction and alkylation were then performed by incubation with 10 mmol·L^−1^ dithiothreitol (DTT; Amresco, China) in 25 mmol·L^−1^ NH_4_HCO_3_ (Macklin, China) at 37°C for 1 hour, followed by dark incubation with 20 mmol·L^−1^ iodoacetamide (Macklin, China) in 25 mmol·L^−1^ NH_4_HCO_3_ for 1 hour. The treated proteins were transferred to an ultrafiltration device (Merck Millipore, USA), washed three times with 50 mmol·L^−1^ NH_4_HCO_3_, and digested with 3 µg of trypsin (Promega, USA) in 50 mmol·L^−1^ NH_4_HCO_3_ buffer (pH 8.0) at 37°C for 16 hours. After digestion, the mixture was centrifuged at 14,000 × g for 15 minutes at 4°C. The resulting peptides were washed twice with 50 mmol·L^−1^ NH_4_HCO_3_ (pH 8.0) to yield the peptide solution. Digestion was terminated by adding formic acid (Thermo Scientific, USA) to a final concentration of 1% (v/v). Finally, the solution was dried using a centrifugal concentrator (LABCONCO, USA).

The resulting peptides were vacuum-dried, reconstituted in 0.1% formic acid (FA) aqueous solution, and quantified via absorbance measurement at 280 nm using a NanoDrop 2000 spectrophotometer. Peptides were then diluted to a working concentration of 500 ng/µL in 0.1% FA aqueous solution. iRT peptides were spiked into each sample as retention time calibration internal standards prior to loading, according to the required injection volume.

Peptide identification was performed using an Easy-nLC 1200 – Orbitrap Fusion mass spectrometer system (Thermo Fisher Scientific, America). Chromatography utilized a PepMap C18 column (75 μm × 25 cm) with mobile phase A (0.1% FA in water) and mobile phase B (0.1% FA in 80% acetonitrile) at a flow rate of 0.3 μL/min. The gradient program was as follows: 0–102 min (5–25% B), 102–110 min (25–38% B), 110–112 min (90% B). Mass spectrometry parameters were: electrospray voltage, 2.3 kV; DIA-MS scan range, 300–1700 m/z; MS1 and MS2 resolutions, 120,000 and 30,000 (at m/z 200), respectively; maximum injection times, 50 ms (MS1) and 54 ms (MS2); 100 fixed isolation windows covering m/z 400–1000.

### SDS-PAGE analysis of protein integrity

Four randomly selected frozen serum samples per group were subjected to total protein concentration determination using the BCA assay (Thermo Fisher Scientific, USA). Aliquots containing 50 µg of total protein were mixed with reducing loading buffer and denatured at 95°C for 10 min. Electrophoresis was performed using 10% separating gels: 80 V constant voltage during the stacking phase, increased to 110 V once the dye front entered the separating gel, and continued until the bromophenol blue dye front reached the gel bottom. Following electrophoresis, gels were stained with Coomassie Brilliant Blue R-250 (Beyotime, China) for 30 min and subsequently destained until protein bands were clearly visible.

### ICP-MS method development and validation

Serum trace metal analysis was performed on 200 long-term and 200 short-term cryopreserved specimens using triple quadruple ICP-MS (Agilent 8800). Precise 0.2 mL serum aliquots were diluted to 10 mL with 1% nitric acid in 15 mL centrifuge tubes, then centrifuged at 3000 rpm for 15 min. Calibration standards (0.1–100 μg/L) in 1% HNO_3_ were prepared from a element mix (Agilent 8500-6940). We analyzed ten metals: vanadium (V), chromiumm (Cr), manganese (Mn), iron (Fe), copper (Cu), zinc (Zn), selenium (Se), rubidium (Rb), strontium (Sr), and cesium (Cs) using germanium (Ge), indium (In), and terbium (Tb) isotopes (Agilent 5188–6525) as internal standards. Instrument parameters were as follows (Table 1).

**Table 1:** Instrumental parameters for serum metal analysis by ICP-MS.

Parameter	Setting Value
Nebulizer gas flow/(L/min)	0.99
Auxiliary gas flow/(L/min)	0.9
Plasma gas flow/(L/min)	15
Helium gas flow/(L/min)	5
Peristaltic pump speed/(r/s)	0.1
Sampling depth/mm	10
RF power/W	1550
Spray chamber temperature/°C	2
Number of replicates	3

### Method validation

The method’s performance was evaluated for linearity, sensitivity, precision, and accuracy. Limits of detection (LOD) and quantification (LOQ) were derived from 15 consecutive measurements of a procedural blank, with calibration curves maintained at r > 0.99. Concentrations below LOQ were substituted with LOQ/√2 for statistical analysis. LOD and LOQ were calculated as follows:


$$\begin{array}{c} LOD=\frac{X_{L}-\overset{―}{X_{b}}}{\text{S}} \end{array}$$

$$\begin{array}{c} LOQ=\frac{\text{10}S_{b}}{\text{S}} \end{array}$$

*S*_*b*_ is the standard deviation of the blank signal, *S* is the calibration slope, and *X*_*L*_ denotes the minimum detectable signal at a given confidence level(*X*_*L*_ = $\overset{―}{X_{b}}$+K*S*_*b*_). The final method detection limit was obtained by multiplying the instrumental LOD by the sample dilution factor of 50.

Precision was assessed at intra‑day and inter‑day levels. Intra‑day precision was determined by analyzing three plasma samples 12 times in one day; inter‑day precision was evaluated by analyzing the same samples over three consecutive days. Results are expressed as relative standard deviation (RSD):


$$\begin{array}{c} RSD=\frac{\sqrt{\sum_{i=\text{1}}^{n}\left(X_{i}-\overset{―}{X}\right)}}{\overset{―}{X}}\times \text{1}\text{0}\text{0}\% \end{array}$$

where n is the number of measurements, $X_{i}$ is an individual concentration, and $\overset{―}{X}$ is the mean concentration.

A spike recovery experiment was conducted to evaluate accuracy. Four 200 μL aliquots of plasma were prepared: one unspiked control and three spiked with low (1 μg/L), medium (5 μg/L), and high (10 μg/L) analyte concentrations. All received 200 μL of a 100 μg/L internal standard solution. Recovery (REC, %) was calculated as:


$$\begin{array}{c} REC=\frac{C_{\text{total}}-C_{\text{sample}}}{C_{\text{spike}}} \end{array}$$

$C_{\text{total}}$ is the measured concentration in the spiked sample, $C_{\text{sample}}$ is the concentration in the unspiked sample, and $C_{\text{spike}}$ is the known spike concentration.

### Sample processing and analysis

Frozen serum samples were thawed at 4°C. A 0.2 mL aliquot was diluted 50‑fold with 1% nitric acid to 10 mL, vortexed, and centrifuged (3000 rpm, 15 min). The clear supernatant was analyzed using the validated ICP‑MS method. Each run included procedural blanks and quality control samples to monitor contamination and ensure analytical accuracy.

### Proteomic data processing and analysis

DIA data were processed using DIA-NN (version 1.8.2) with the following parameters: Homo sapiens proteome; tryptic digestion (≤2 missed cleavages); false discovery rate (FDR) ≤ 0.01. Precursor selection was based on deep learning-based spectra and retention time prediction, with peptide lengths ranging from 7 to 30 amino acids and a precursor scan range of 300–1800 m/z. All other parameters were set to their default values. Missing values were imputed using random values drawn from a Gaussian distribution centered below the median, as implemented in the Perseus software [17].

Gene Ontology (GO) and KEGG pathway analyses were conducted via bioinformatics.com.cn (accessed May 15, 2025) using cluster Profiler and path view R packages Protein identification counts, peptide identification counts, and coefficients of variation (CV) for precursor ion peak intensities within groups were analyzed using Microsoft Excel 2016. CV density plots, missing value matrix plots, and volcano plots were generated via the OmicStudio cloud platform (https://www.omicstudio.cn) [18].


$$\begin{array}{c} CV=\left(\frac{\sigma }{\mu }\right)\times \text{1}\text{0}\text{0}\% \end{array}$$

σ represents the standard deviation, and *µ* denotes the mean.

### Statistical analysis

Analyses used SPSS (v25.0). Continuous variable normality was assessed with the Kolmogorov-Smirnov test. Normally distributed data appear as mean ± SD, non-normally distributed data as median (Q1-Q3). Categorical variables were compared using χ² tests. Non-parametric tests evaluated group differences for non-normal variables. Statistical significance was set at *P* < 0.05.

To determine whether storage duration remained an independent predictor of serum metal concentrations after accounting for demographic differences, multiple linear regression analyses were performed for metals that showed significant differences in unilabiate comparisons. The primary independent variable was storage group (long-term vs. short-term), and age, BMI, and sex were included as covariates in the model. Unstandardized regression coefficients (B) with 95% confidence intervals and standardized coefficients (β) are reported. Statistical significance was set at *P* < 0.05.

### Metal correlation analysis

The influence of long-term cryopreservation on the intrinsic correlation patterns of metallic elements was assessed by performing Spearman rank correlation analysis on the metal concentration data from samples archived in 2018 and 2023. Correlation heatmaps, generated using GraphPad Prism 9.0 (with α = 0.05), were employed to visualize and compare the structural changes in the metal correlation networks between these two time points.

## Results

### Identification of proteins

**Fig 1:**
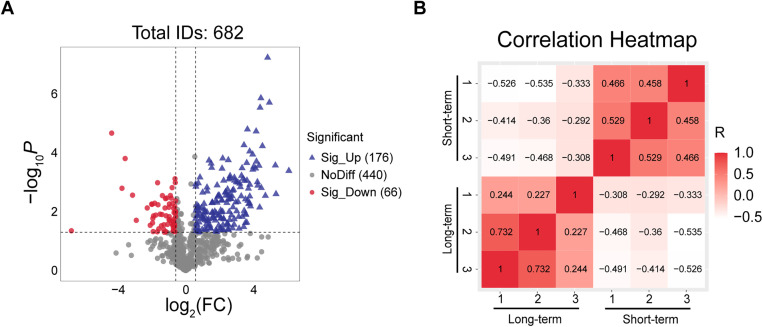
Identification of differentially proteins associated between long-term cryopreservation and short-term cryopreservation. **(A)** Volcano plot for the comparison between the long-term cryopreservation and short-term cryopreservation. The cutoff values *p*-Val < 0.05 and FC > 1.5 were utilized to identify differentially expressed proteins. Red color is indicative of up-regulate proteins and blue color is indicative of down regulated proteins. **(B)** Correlation matrix for all 6 samples. The intensity of the color reflects the strength of the correlation, with darker shades representing higher correlations.

Three biological replicate pools from each group (long-term and short-term cryopreservation) were analyzed using the Orbitrap Fusion mass spectrometry platform. *P*-values for protein fold changes were calculated using a permutation test via the OmicStudio platform (https://www.omicstudio.cn/tool). Proteins with FC > 1.5 were considered significantly altered upon long-term cryopreservation. Among the 682 quantified proteins, 242 proteins were statistically significant, unsupervised hierarchical clustering analysis revealed 176 up-regulated proteins and 66 down- regulated proteins in long-term cryopreservation compared to those in short-term cryopreservation (Fig 1A). The quantification precision of proteins across the three replicates in this study was compared by plotting the intensity of each protein group in one run against another, as shown in Fig 1B. Pearson correlation coefficients were high within groups and low between groups. The results indicated the reliability and high quality of the proteomic identification data in this study.

### LC-MS/MS data quality assessment

**Fig 2:**
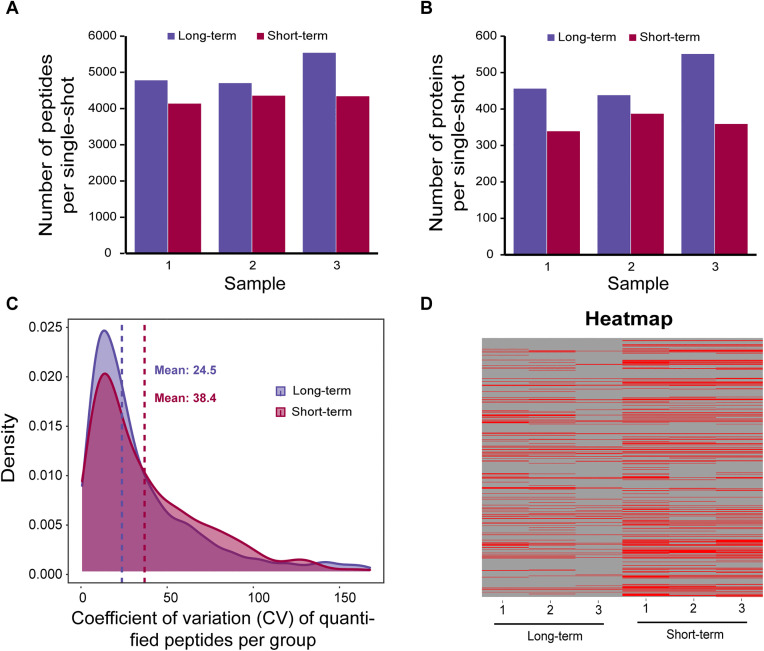
Proteomics data quality assessment. **(A)** Single-injection peptide identification count comparison. **(B)** Single-injection protein identification count comparison.**(C)** Density distribution of precursor ion peak intensity CV. **(D)** Missing value matrix for identified proteins between groups, with red lines indicating missing values.

LC-MS/MS data quality was evaluated based on protein identification counts, peptide identification counts, CV, and missing values across both sample groups. The long-term cryopreservation samples yielded higher numbers of identified proteins and peptides compared to the short-term cryopreservation samples(Fig 2A–B). The mean CV values for the two groups were 24.5% and 38.4%, respectively(Fig 2C). Lower CV values for peptide peak intensities within a group indicate higher quantitative reproducibility. These results collectively demonstrate superior data reproducibility in the long-term cryopreservation serum samples relative to the short-term cryopreservation samples.

Missing value rates within each group were 15% and 29%, respectively. As illustrated in Fig 2D, the data matrix heat map visually depicts missing data distribution (indicated in red), with substantially less red area in the long-term cryopreservation group compared to the short-term group. This confirms higher data completeness in the long-term cryopreservation samples.

### Protein ontology (go) analysis and KEGG pathway analysis

**Fig 3:**
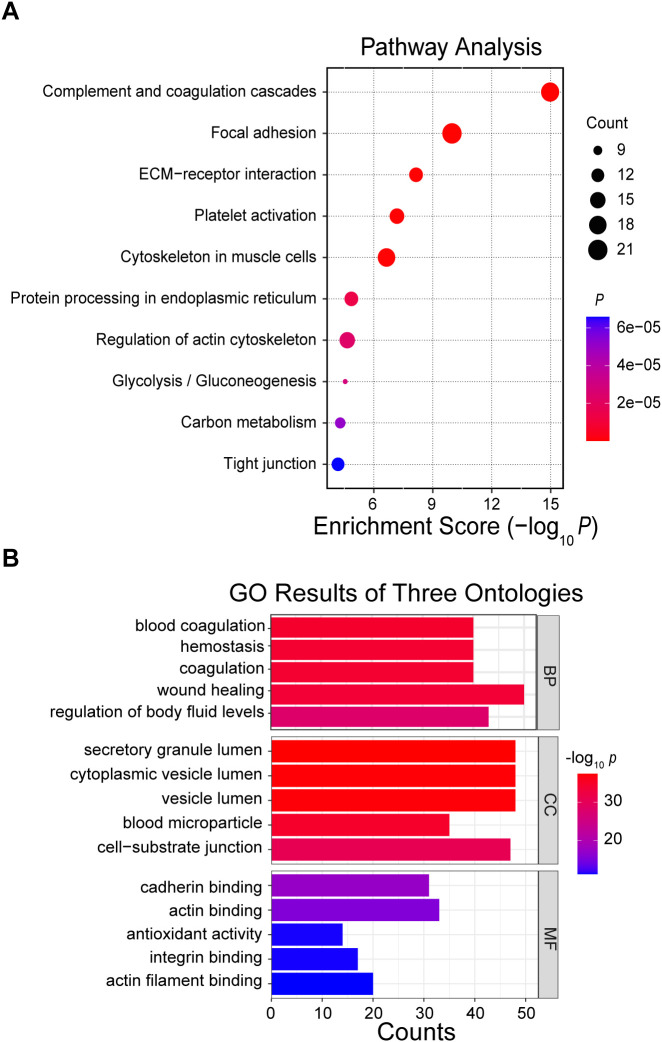
GO enrichment and KEGG pathway analysis of differentially samples from long-term cryopreservation vs short-term cryopreservation. **(A)** Differentially protein GO enrichment analysis. The x-axis represents the number of proteins in the pathway, while the y-axis represents the pathway names. The color scale indicates the enrichment score of the *p*-value. **(B)**The horizontal axis represents the Enrichment Score in the pathway, while the vertical axis represents the pathway name. The color scale indicates different thresholds of the *p*-value, and the size of the dot indicates the number of proteins corresponding to each term.

To determine the signaling pathways associated with long-term cryopreservation, GO enrichment analysis KEGG pathway analysis was conducted for all identified proteins using online tools (https://www.bioinformatics.com.cn). The findings demonstrate significant alterations in protein expression profiles related to immune responses, metabolic pathways, extracellular matrix (ECM) organization, and antioxidant activity in serum samples cryopreserved for six years. Notably, pathways including the complement and coagulation cascades, ECM-receptor interactions, and glycolysis/gluconeogenesis exhibited significant enrichment in the long-term cryopreservation. This suggests that long-term storage may compromise the stability or modify the activity of specific immune factors, metabolic enzymes, and structural proteins. Furthermore, substantial changes were observed in Gene Ontology (GO) terms associated with blood coagulation, antioxidant activity, and cytoskeletal organization, supporting the hypothesis that prolonged cryopreservation induces protein degradation or oxidative stress. Consequently, long-term cryopreservation may substantially influence proteomic analysis outcomes, particularly for metabolism- and immunity-related proteins. This study underscores the necessity for stringent control of cryopreservation duration during serum sample processing or explicit consideration of storage time effects during data interpretation. (Fig 3A, B).

### SDS-PAGE analysis of protein integrity

**Fig 4:**
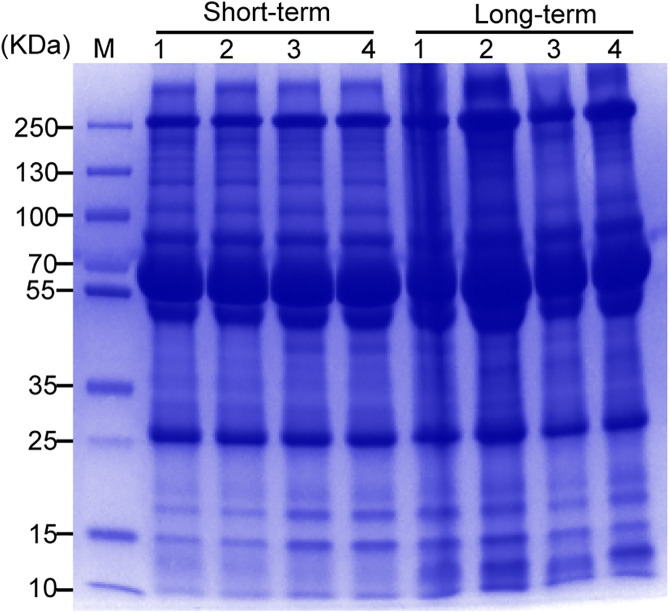
SDS-PAGE analysis of serum proteins after short‑term and long‑term cryopreservation.

SDS-PAGE analysis revealed clear differences in serum protein banding patterns between long-term and short-term cryopreserved samples. In the high-molecular-weight region (>100 kDa), band intensities were markedly reduced in long-term samples, accompanied by pronounced smearing and the near disappearance of discrete bands. At approximately 250 kDa, staining intensity was substantially higher in long-term samples, suggesting the accumulation of high-molecular-weight protein aggregates. In contrast, the low-molecular-weight region (<35 kDa) showed both increased band number and enhanced staining intensity, reflecting progressive accumulation of small degradation products. This reciprocal pattern (Fig 4).

### Validation of ICP-MS method

During the analytical process, all calibration curves demonstrated correlation coefficients greater than 0.999, indicating excellent linearity. Consequently, the intra-day precision for each element under this method was better than 10%, while the inter-day precision for most elements was also better than or approached 20%. Furthermore, spike recovery results showed that the recovery rates for all elements at low, medium, and high concentration levels fell within 70% to 120%, with average recoveries ranging from 85% to 115%. Therefore, this method is deemed suitable for the detection of these ten metallic elements (S1–S3 Tables).

### Metallome analysis

We quantified ten serum elements (V, Cr, Mn, Fe, Cu, Zn, Se, Rb, Sr, Cs) by ICP-MS to assess long-term cryopreservation effects. Baseline characteristics appear in Table 2. Gender distribution showed no significant difference between long- and short-term cryopreserved groups (*P* > 0.05). However, the long-term group had significantly higher BMI and lower age (*P* < 0.05).

**Table 2:** General information of the volunteers.

Group	N of cases	Sex		Age(year)	BMI (kg/m^2^)
		Male (%)	Female (%)		
2023	200	100 (50)	100 (50)	26.81 ± 4.42	21.11 ± 2.52
2018	200	100 (50)	100 (50)	25.72 ± 9.72	21.63 ± 2.11
Statistic		0.160^a^		4.09^b^	−3.436^b^
*P*		0.689		0.000052	0.001

Note: a = χ² (chi-square); b = t-value.

Kolmogorov-Smirnov tests indicated non-normal distributions for all elements, persisting after log-transformation. Elemental concentrations are therefore reported as median (IQR). Significant differences (*P* < 0.05) emerged for V, Mn, Fe, Zn, Rb, and Cs between preservation durations (Table 3).

**Table 3:** Comparison of the serum from healthy adults between long-term and short-term cryopreservation.

Group	V	Cr	Mn	Fe	Cu
2023	1.58 (1.15, 2.02)	2.03 (1.37, 3.31)	2.99 (2.39, 3.68)	881.35 (665.27, 1154.68)	755.68 (669.35, 876.63)
2018	1.12 (0.82, 1.47)	2.35 (1.82, 2.81)	0.84 (0.49, 1.18)	1183.51 (877.71, 1464.47)	785.87 (671.27, 895.66)
Z	−6.934	−1.922	−15.135	−6.25	−1.332
*P*	4.0834E-12	0.055	9.5695E-52	4.1021E-10	0.183
**Group**	**Zn**	**Se**	**Rb**	**Sr**	**Cs**
2023	679.99 (605.75, 775.14)	92.66 (71.56, 109.25)	146.99 (129.27, 165.58)	28.28 (23.86, 33.95)	0.74 (0.58, 0.96)
2018	942.70 (831.63, 1063.73)	86.36 (66.17, 114.60)	376.69 (308.52, 483.35)	27.35 (22.58, 32.19)	1.14 (0.87, 1.46)
Z	−13.454	−0.714	−17.163	−1.393	−9.575
*P*	2.908E-41	0.475	5.0162E-66	0.164	1.0192E-21

### Multivariate adjustment for potential confounders

After adjustment, long-term storage remained a highly significant independent predictor for six of the ten metals examined. Specifically, long-term storage was associated with significantly lower concentrations of V and Mn, and significantly higher concentrations of Fe, Zn, Rb, and Cs (Table 4). Among the covariates, age showed a modest positive association with V (β = 0.027, P = 0.014) and a modest negative association with Rb (β = −5.777, *P* = 0.015), while BMI and sex did not exhibit consistent or significant associations with most metal concentrations. These findings confirm that the alterations in V, Mn, Fe, Zn, Rb, and Cs are robust to demographic adjustment and are primarily attributable to the duration of cryopreservation.

**Table 4:** Independent effect of long-term cryopreservation on serum metal concentrations after adjustment for age, BMI, and sex.

Metal	Unstandardized B (95% CI)	Standardized β	P-value
V	−0.399 (−0.516, −0.282)	−0.325	<0.001
Mn	−2.124 (−2.35, −1.90)	−0.7	<0.001
Fe	312.877 (213.90, 411.81)	0.307	<0.001
Zn	260.386 (224.95, 295.68)	0.605	<0.001
Rb	266.346 (241.22, 291.47)	0.723	<0.001
Cs	0.416 (0.324, 0.507)	0.422	<0.001

### Metal correlation analysis

**Fig 5:**
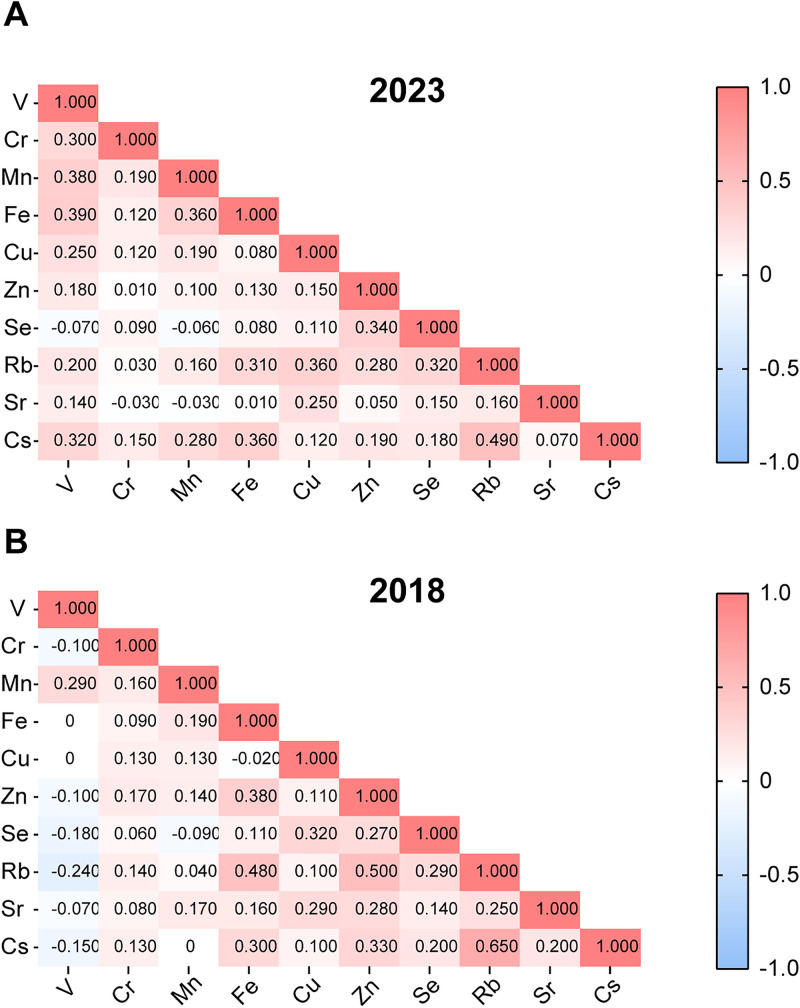
Impact of long-term cryopreservation on element correlations in plasma samples. **(A)** Correlation matrix of metal elements in serum samples from the 2023 Shenzhen Chronic Disease and Risk Factor Surveillance Project. **(B)** Correlation matrix of metal elements in serum samples from the 2018 Shenzhen Chronic Disease and Risk Factor Surveillance Project. Color intensity represents the magnitude of the correlation coefficient, with red indicating positive correlations and blue indicating negative correlations.

This study investigated the impact of long-term cryopreservation on correlations among plasma metallic elements. Pairwise correlations of ten metals were compared using serum samples obtained from the Shenzhen Chronic Disease and Risk Factor Surveillance Project in 2018 and 2023. The results demonstrated that prolonged storage significantly altered the correlation structure. Correlations involving vanadium (V) weakened or became negative: V–Fe decreased from 0.391 to nearly zero, V–Cr from 0.300 to –0.100, V–Rb from 0.200 to –0.240, and V–Cs from 0.320 to –0.150. New correlations also emerged following long-term storage, including moderate positive correlations for Fe–Zn (r = 0.38), Fe–Rb (r = 0.48), and Zn–Rb (r = 0.50). In contrast, these pairs exhibited only weak correlations in short-term preserved samples (Fig 5A and 5B).

## Discussion

This study systematically evaluated the impact of cryopreservation duration on the concentrations of 10 metallic elements and their intrinsic correlations by comparing serum samples subjected to short-term and long-term low-temperature storage. The key findings reveal that long-term cryopreservation is not a homogeneous process; it significantly alters the concentrations of multiple metals, and reshapes the serum metal correlation network. These conclusions serve as an important caution for retrospective studies based on biobanked samples.

Quantitative proteomics identified 242 differentially abundant proteins, of which approximately 2.7 times more were upregulated than downregulated. LC-MS/MS data indicated that long-term preserved samples exhibited artifacts of over-optimization, including increased peptide/protein identifications, lower coefficients of variation (24.5% vs. 38.4%), and fewer missing values (15% vs. 29%). These trends likely resulted from interference by degradation products. Together, the findings confirm that prolonged cryostorage compromises molecular integrity via physical degradation, pathway dysregulation, and elemental migration.

The molecular changes observed here can be explained by established mechanisms. Temperature fluctuations during cryopreservation critically affect metabolite and small molecule stability, partly by reactivating metabolic enzymes and promoting oxidative degradation of temperature-sensitive compounds [19]. Protein denaturation occurs at ice–liquid interfaces where pH fluctuates, whereas slow freezing (>4 h) can induce aggregation [20]. These conditions accelerate the degradation of compounds such as adenosine and disulfides through molecular crowding and pH shifts. For some proteins, low temperature alone is sufficient to induce conformational changes [21,22]. In addition, freeze concentration can trigger secondary reactions—including aggregation, crystallization of buffer salts, phase separation, and solute redistribution—all of which jeopardize protein stability [23].

Notably, Savickas and colleagues have shown that C-terminomics and N-terminomics can capture transient intermediates and cleavage sites produced during protein degradation—information that intact proteins simply do not provide [24]. During storage, protein degradation exposes cryptic protease cleavage sites and generates peptide intermediates absent from fresh samples. Upon trypsin digestion, these degradation-derived species enter the detection workflow and artificially inflate the number of identifiable peptides and proteins. This shift is reflected in a smaller CV, as smaller peptides tend to homogenize chromatographic retention time drift and ionization variability, and in fewer missing values, due to their higher solubility and easier capture. The gain in identification depth is driven both by the greater abundance of fragments from degradation and by the higher ionization efficiency of small peptides. These observations are consistent with earlier reports that degraded peptides can contribute to false‑positive identifications [25,26].

Trace element analysis further supports the observed degradation, revealing systematic concentration shifts in V, Mn, Fe, Zn, Rb, and Cs within long-term cryopreserved serum. Three underlying mechanisms drive these changes. Ice crystal formation physically disrupts cellular and protein structures, releasing bound intracellular elements such as Fe and Zn into the serum. Meanwhile, cold denaturation of antibody proteins exposes hydrophobic regions and alters tertiary structure, reducing their metal-binding capacity [27,28]. In addition, V and Mn—which primarily associate with high-molecular-weight proteins like transferrin and α_2_-macroglobulin—decline in soluble form as these proteins aggregate and precipitate over time [29,30]. Adsorption further contributes to loss, as silicone-based groups on polypropylene cryotube surfaces exhibit high affinity for high-valence ions like V^5+^ and Mn^3+^ [31–33].

Furthermore, we observed a marked weakening or reversal of correlations between vanadium (V) and several other elements following long-term cryopreservation. This phenomenon may be linked to the redox sensitivity and labile protein-binding characteristics of vanadium in biological matrices. Notably, previous studies report that vanadium in plasma is predominantly bound to transferrin; hence, prolonged storage could induce protein conformational alterations or partial degradation, thereby perturbing its co-transport dynamics or competitive equilibria with other metals [34,35].Concurrently, novel moderate positive correlations emerged after extended storage among Fe–Zn, Fe–Rb, and Zn–Rb pairs. These emergent associations likely indicate a redistribution or co-segregation of specific metals within the sample matrix during long-term cryopreservation, potentially driven by shared physicochemical properties such as comparable hydrated ionic radii, coordination chemistry, or common biological transport pathways [9,36]. Collectively, these results imply that long-term cryopreservation not only affects absolute metal concentrations but, more critically, reshapes the multivariate correlational architecture, leading to a partial dismantling or reorganization of the inherent elemental interaction network. Consequently, researchers should exercise caution to account for potential systematic biases introduced by storage duration in retrospective multi-element or metabolomic pattern analyses utilizing biobanked samples.

Several limitations should be considered. First, although metallomic findings were robust to adjustment for age, BMI, and sex, the proteomic analysis used pooled samples and could not be adjusted at the individual level. Second, the small number of biological replicates (three pools per group) limits statistical power, and the differentially expressed proteins should be viewed as exploratory candidates. Third, undetected protein oxidation could lead to underestimating true cryodamage.Future studies employing individual-level proteomics with larger sample sizes, longitudinal sampling designs, isotope tracing for adsorption validation, and multivariate models integrating proteomic and metallomic data are warranted to extend these findings. Finally, defining cryostorage time limits for different biomolecules would offer practical guidelines for biobanking operations.

## Supporting information

S1 Table Correlation coefficient, linear range, and limits of detection for each element. (DOCX)

S2 Table Intra-day and Inter-day precision results of elements. (DOCX)

S3 Table Spike recovery rates for each element. (DOCX)

S4 Table Full multiple linear regression results for all serum metals. (DOCX)

S1 File SDS-PAGE raw images. (PDF)
