# Supplementary material for: Impact of long-term cryopreservation on serum proteome and metallome: Implications for Biobank quality control
Source: PLoS One. 2026 Jun 25;21(6):e0351736. doi: 10.1371/journal.pone.0351736 (PMC13298790; doi:10.1371/journal.pone.0351736)
Supplement: S1 Table — (DOCX) [file pone.0351736.s001.docx]

**S1 Table 1.** Correlation Coefficient, Linear Range, and Limits of Detection for Each Element

| Element | Isotope | Correlation Coefficient  (*R^2^*) | Linear Range  (μg/L) | Instrumental LOD (μg/L) | Method LOD (μg/L) |
| --- | --- | --- | --- | --- | --- |
| V | 51 | 0.9995 | 0.1-10 | 0.0045 | 0.23 |
| Cr | 52 | 0.9990 | 0.1-10 | 0.0125 | 0.63 |
| Mn | 55 | 0.9997 | 0.1-50 | 0.0062 | 0.31 |
| Fe | 56 | 0.9997 | 0.1-50 | 0.0302 | 1.51 |
| Cu | 63 | 0.9996 | 0.1-50 | 0.0072 | 0.36 |
| Zn | 66 | 0.9991 | 0.1-50 | 0.2869 | 14.35 |
| Se | 78 | 0.9980 | 0.1-50 | 0.2878 | 14.39 |
| Rb | 85 | 0.9997 | 0.1-20 | 0.0121 | 0.61 |
| Sr | 88 | 1 | 0.1-5 | 0.0089 | 0.44 |
| Cs | 133 | 0.9991 | 0.1-2 | 0.0031 | 0.15 |
