# Supplementary material for: Impact of long-term cryopreservation on serum proteome and metallome: Implications for Biobank quality control
Source: PLoS One. 2026 Jun 25;21(6):e0351736. doi: 10.1371/journal.pone.0351736 (PMC13298790; doi:10.1371/journal.pone.0351736)
Supplement: S3 Table — (DOCX) [file pone.0351736.s003.docx]

**S3 Table.** Spike Recovery Rates for Each Element

| No | Element | Intra-day Precision (%) | Inter-day Precision (%) |
| --- | --- | --- | --- |
| 1 | V | 4.13 | 8.85 |
| 2 | Cr | 3.06 | 38.11 |
| 3 | Mn | 2.10 | 11.09 |
| 4 | Fe | 2.65 | 7.68 |
| 5 | Cu | 1.56 | 22.95 |
| 6 | Zn | 5.74 | 11.84 |
| 7 | Se | 2.53 | 9.61 |
| 8 | Rb | 5.88 | 26.00 |
| 9 | Sr | 2.52 | 13.77 |
| 10 | Cs | 6.31 | 23.76 |
