# Supplementary material for: Impact of long-term cryopreservation on serum proteome and metallome: Implications for Biobank quality control
Source: PLoS One. 2026 Jun 25;21(6):e0351736. doi: 10.1371/journal.pone.0351736 (PMC13298790; doi:10.1371/journal.pone.0351736)
Supplement: S4 Table — (DOCX) [file pone.0351736.s004.docx]

**S4 Table. Full multiple linear regression results for all serum metals.**

| **Metal** | **Predictor** | **Unstandardized B (95% CI)** | **Standardized β** | ***P*-value** |
| --- | --- | --- | --- | --- |
| **V** | Group | -0.399 (-0.516, -0.282) | -0.325 | <0.001 |
|  | Age | 0.027 (0.006, 0.049) | 0.121 | 0.014 |
|  | BMI | 0.001 (-0.037, 0.038) | 0.002 | 0.967 |
|  | Sex | 0.117 (0.001, 0.232) | 0.095 | 0.048 |
| **Mn** | Group | -2.124 (-2.35, -1.90) | -0.700 | <0.001 |
|  | Age | -0.012 (-0.054, 0.029) | -0.022 | 0.551 |
|  | BMI | -0.019 (-0.090, 0.053) | -0.019 | 0.603 |
|  | Sex | -0.046 (-0.267, 0.175) | -0.015 | 0.681 |
| **Fe** | Group | 312.877 (213.90, 411.81) | 0.307 | <0.001 |
|  | Age | -1.901 (-20.171, 16.354) | -0.010 | 0.837 |
|  | BMI | 13.563 (-18.134, 45.256) | 0.041 | 0.401 |
|  | Sex | -58.269 (-156.289, 39.532) | -0.057 | 0.242 |
| **Zn** | Group | 260.386 (224.95, 295.68) | 0.605 | <0.001 |
|  | Age | 1.296 (-5.230, 7.822) | 0.016 | 0.696 |
|  | BMI | -7.283 (-18.610, 4.043) | -0.052 | 0.207 |
|  | Sex | 20.696 (-14.289, 55.682) | 0.048 | 0.246 |
| **Rb** | Group | 266.346 (241.22, 291.47) | 0.723 | <0.001 |
|  | Age | -5.777 (-10.416, -1.137) | -0.085 | 0.015 |
|  | BMI | 1.168 (-6.883, 9.220) | 0.010 | 0.776 |
|  | Sex | -23.965 (-48.834, 0.904) | -0.065 | 0.059 |
| **Cs** | Group | 0.416 (0.324, 0.507) | 0.422 | <0.001 |
|  | Age | -0.009 (-0.026, 0.008) | -0.051 | 0.286 |
|  | BMI | -0.010 (-0.040, 0.019) | -0.032 | 0.491 |
|  | Sex | 0.004 (-0.087, 0.094) | 0.004 | 0.939 |
