# Supplementary figures and images for: Impact of long-term cryopreservation on serum proteome and metallome: Implications for Biobank quality control
Source: PLoS One. 2026 Jun 25;21(6):e0351736. doi: 10.1371/journal.pone.0351736 (PMC13298790; doi:10.1371/journal.pone.0351736)

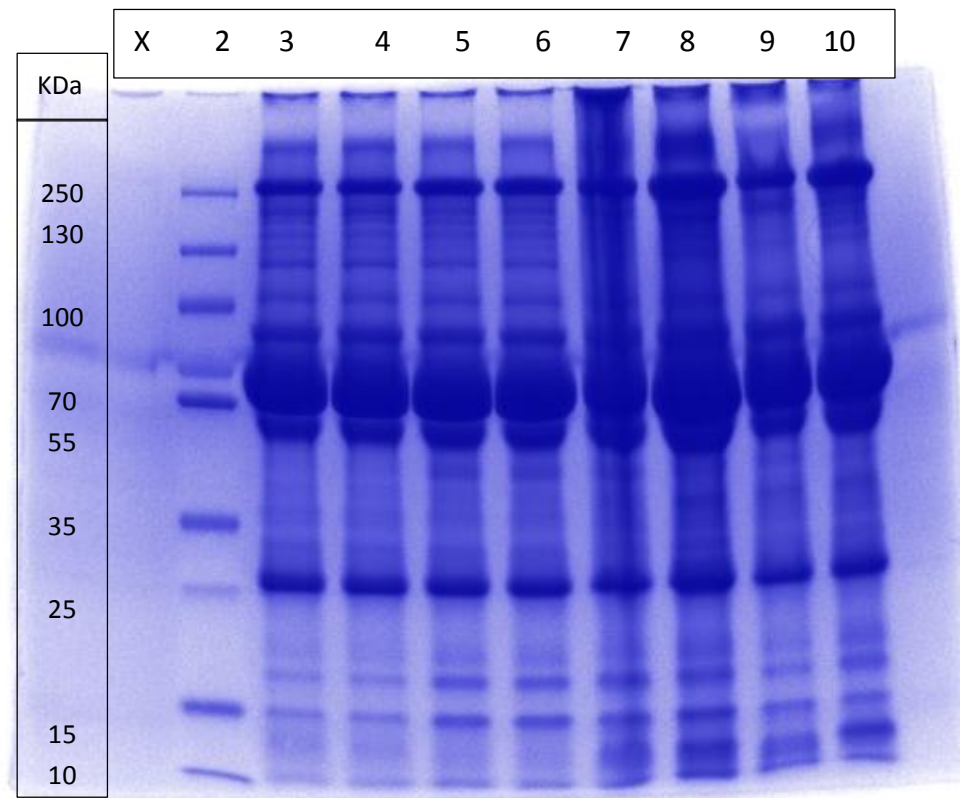

Supplement: S1 File — (PDF) [file pone.0351736.s005.pdf]
